# *Henipavirus* Infection in Fruit Bats (*Pteropus giganteus*), India

**DOI:** 10.3201/eid1408.071492

**Published:** 2008-08

**Authors:** Jonathan H. Epstein, Vibhu Prakash, Craig S. Smith, Peter Daszak, Amanda B. McLaughlin, Greer Meehan, Hume E. Field, Andrew A. Cunningham

**Affiliations:** * The Consortium for Conservation Medicine, New York, New York, USA; † Bombay Natural History Society, Mumbai, India; ‡ Department of Primary Industries and Fisheries, Brisbane, Queensland, Australia; § Cummings School of Veterinary Medicine at Tufts University, North Grafton, Massachusetts, USA; ¶ Australian Animal Health Laboratory, Geelong, Victoria, Australia; # Zoological Society of London, London, UK

**Keywords:** Henipavirus, Nipah virus, Hendra virus, *Pteropus giganteus*, fruit bat, India, flying fox, encephalitis, South Asia, dispatch

## Abstract

We tested 41 bats for antibodies against Nipah and Hendra viruses to determine whether henipaviruses circulate in pteropid fruit bats (*Pteropus giganteus*) in northern India. Twenty bats were seropositive for Nipah virus, which suggests circulation in this species, thereby extending the known distribution of henipaviruses in Asia westward by >1,000 km.

Nipah virus (NiV) and Hendra virus (HeV) are zoonotic paramyxoviruses (genus *Henipavirus*) that have caused human deaths in Australia, Malaysia, Singapore, India, and Bangladesh (*1*–*4*). Known reservoirs for henipaviruses are *Pteropus* spp. fruit bats, which are distributed across the Indo-Pacific region from Madagascar eastward to the South Pacific islands (*5*). Evidence of henipavirus infection has been reported in *Pteropus* bats from Malaysia, Bangladesh, Australia, Thailand, Cambodia, Indonesia, and Madagascar, which supports the theory that these bats have co-evolved with henipaviruses (*6*–*8*).

The first known outbreak of NiV encephalitis in India occurred in 2001 in Siliguri, West Bengal (*1*). The fruit bat (*P. giganteus*) is present across the Indian subcontinent and, although it is suspected as the reservoir host for NiV in Bangladesh, its status as a reservoir for henipaviruses in India is unknown. Seven outbreaks of NiV encephalitis were recognized in Bangladesh from 2000 through 2008, and antibodies to NiV have been found in *P. giganteus* in several colonies there, including colonies adjacent to human case-patients (*3*,*5*,*9*). In the current study, we examined a population of *P. giganteus* bats in India, >1,000 km west of Siliguri, for antibodies to henipaviruses.

## The Study

We captured 41 *P. giganteus* bats from a colony in Haryana State in northern India from June 24 through June 30, 2003, by using mist nets. Blood was collected from the brachial or cephalic artery or from the vein by using a heparinized 3.0-mL syringe and a 22-gauge or 27-gauge needle and stored for 24 hours at 4°C to allow for plasma separation; the separated plasma was then stored at –20°C until use. Sex, age, body condition score, pregnancy status, lactation status, weight, and forearm length were recorded. Age was estimated by the presence of secondary sexual characteristics and dental wear. Body condition was assessed by digital palpation of the pectoral muscles and individuals were assigned a body condition score (BCS) of “poor,” “fair,” or “good.” Unweaned juveniles were not assigned a BCS because of their physical immaturity. Pregnancy was determined by digital palpation, and a bat was considered “lactating” if milk could be expressed from either teat. All bats were released after sampling.

All 41 plasma samples were screened for antibodies to NiV and HeV by using virus-specific indirect ELISAs. Thirty-nine samples (2 samples had insufficient amounts of plasma remaining) were analyzed by using NiV and HeV serum neutralization tests (SNTs) under Biosafety Level 4 conditions (*10*). For the ELISA, coating antigen was derived from purified HeV- and NiV-infected Vero cells, and positive control serum specimens were obtained from experimentally infected horses (HeV) and pigs (NiV). Protein A/G conjugate was used to detect bound bat serum. A final serum dilution of 1:50 was used for the bat samples. A sample was considered reactive if the ratio of its average optical density at 450 nm (OD_450_) of infected Vero cell antigen-coated wells (each sample was tested in duplicate) to uninfected Vero cell antigen-coated wells was >2.0 and the average OD_450_ value for the sample in the infected Vero cell antigen-coated wells was >0.2. Positive control serum samples were confirmed by both ELISA and SNT. SNT results were considered positive if virus neutralization occurred at >1:5 dilution (*11*). If neutralizing antibodies were present for both HeV and NiV, the higher titer was considered the positive test only if the difference between them was >4-fold (*11*). Samples that had positive titers to both viruses that differed by <4-fold were considered positive for an unspecified henipavirus.

The results of the serologic tests are presented in the Table, including comparisons of the results by gender, lactation status (females), and BCS. Twenty-six (63%) of 41 samples (95% confidence interval [CI] 47%–78%) were reactive in the NiV ELISA, 5 of which were also reactive in the HeV ELISA. No plasma samples reacted only in the HeV ELISA. Twenty (51%) of 39 samples (95% CI 35%–68%) had neutralizing antibodies to NiV, and 10 (26%) of 39 (95% CI 13%–42%) had neutralizing antibodies to HeV. One (3%) of 39 samples (95% CI 0%–13%) had a neutralizing titer of 5 to NiV and HeV. This sample reacted in the NiV ELISA, but not in the HeV ELISA, although because it had equivalent neutralizing titers to both viruses, it was considered positive for an unspecified henipavirus. The ELISA showed 95% sensitivity and 75% specificity compared with the SNT.

**Table:** ELISA and SNT results and univariate associations between serostatus and other variables for wild-caught *Pteropus giganteus* bats in India*

Characteristic	ELISA			SNT	NiV SNT comparisons, p value†
	No. NiV reactive/ no. tested	No. HeV reactive/ no. tested		No. NiV positive/total (%) [median titer; range]	
Total	26/41	5/41		20/39‡ (51) [80; 5–640]	
Male	10/12	3/12		8/12 (67) [60; 20–640]	0.300
Female	16/29	2/29		12/27‡ (44) [80; 5–640]	
Lactating	12/20	2/20		8/19‡ (42) [80;20–640]	1.00
Nonlactating	4/9	0/9		4/8‡ (50) [80;5–80]	
Body condition score§					
Poor	5/9	0/9		1/9 (11) [640; NA]	P v F: 0.005; F v G: 0.315; P v G: 0.505
Fair	16/24	5/24		16/23 (70) [80; 5–640]	
Good	3/6	0/6		2/5 (40) [60; 40–80]	

*SNT, serum neutralization test; NiV, Nipah virus; HeV, Hendra virus; NA, not applicable; P, poor; F, fair; G, good. †Fisher exact test p value significant at <0.05. ‡Two samples had insufficient plasma for SNT (both were ELISA negative); sample 1 was from a nonlactating adult female with a good body condition score (BCS) and the other was from a lactating adult with a fair BCS. A third sample, a nonlactating adult female with a good BCS had equivocal NiV/HeV SNT titers (*5*), which was attributed to an unspecified henipavirus and considered negative for NiV and HeV. §Two pre-weaned pups (1 male, NiV SNT negative; 1 female, NiV SNT positive titer 80) were excluded from the BCS dataset because of their physical immaturity.

Each of 2 unweaned pups matched their mother’s serostatus, with 1 pup positive by SNT (pup 80, mother >640). Samples of the other mother–pup pair were seronegative. There were no significant differences in the NiV seroprevalence in male bats on SNT (8/12) compared to female bats (12/27) by using a Fisher exact test (FET; p = 0.300) or in lactating female bats (8/19) compared to nonlactating (5/8) female bats (FET; p = 0.420). We found significant differences in seroprevalence between bats with a poor and fair BCS (FET; p = 0.005), with bats in poor condition having a lower antibody prevalence than those with fair BCS. No difference in seroprevalence was found between the poor and good BCS groups or the fair and good groups.

## Conclusions

Our study provides evidence that NiV, or a closely related henipavirus, circulates in Indian fruit bats (*P. giganteus*), thereby extending the range of the genus *Henipavirus* in Asia westward by >1,000 km. Our results are consistent with reports of NiV in *P. giganteus* bats in Bangladesh (*3*) and with *Pteropus* spp. being the primary reservoir of henipaviruses (*5*). Logistical limitations prevented us from attempting virus isolation and testing for viral RNA.

Previous studies have demonstrated that ELISAs, although less specific than SNTs, are useful screening tests for henipaviruses (*11*). Our results support this assertion, with the ELISA showing a high sensitivity. In our study, neutralizing antibodies to HeV and NiV were detected in 11 bats, 10 of which exhibited a >4-fold titer to NiV antibodies. Concurrent HeV and NiV titers are considered due to cross-neutralization rather than exposure to both viruses (*6*,*11*,*12*). Serologic studies provide information about the proportion of a population exposed to NiV, but not about the prevalence of bats that may be shedding virus or the virus itself. Further work in this area is required to fully characterize the henipavirus(es) involved and to confirm the status of *P. giganteus* as a reservoir.

Researchers have suggested that pregnancy plays a key role in henipavirus transmission among Australian *Pteropus* spp. and from bats to other species (*13*,*14*). In our study, we found no significant difference in seroprevalence between sexes, or between lactating and nonlactating females. Of the 2 lactating females carrying pups, 1 had a high titer of >640 and its pup had a titer of 80 against NiV, which suggests the passive transfer of antibodies; the other dam–pup pair was seronegative. Seroprevalence appeared to be significantly greater in bats with fair BCS when compared with those with poor BCS; however, no significant differences were found between good and poor or good and fair BCS groups. The findings that bats with fair BCS had a higher seroprevalence than poor BCS bats, but that there was no difference between good BCS bats and the other 2 groups, may be explained by the subjective classification of a bat’s body condition. Those bats deemed to have fair body condition may have been more similar to those with robust bodies (good BCS) than those with thin, emaciated bodies (poor BCS). In fact, if one combines the good and fair categories, and compares the seroprevalence (18/28) with that of the poor group, the difference is still significant (p = 0.007); by contrast, combining the fair and poor categories (17/32) and comparing that seroprevalence to the good category results in no significant difference (p = 0.660). Having a lower seroprevalence in bats with the poorest BCS may be explained as an artifact of the nonrandom sampling (we sampled those bats that were first to be captured), by the limited sample, or it could suggest that NiV infection causes death in *P. giganteus* bats that are in poor physical condition. The latter explanation is less plausible because experimental infections of *Pteropus* spp. with henipaviruses produce only subclinical infection with no illness or death (*15*).

In northern India, as in Bangladesh, *P. giganteus* bats live in close association with the human population. Indeed, the colony examined in this study lives in a busy town above a major tourist attraction. Previous studies of NiV encephalitis outbreaks in Bangladesh have identified fresh date palm juice or fruit as plausible foodborne routes of transmission between bats and humans (*3*,*16*). The multiple outbreaks of NiV in Bangladesh, and the 2001 outbreak in West Bengal, show a continued risk for spillover infection between bats and humans in this region. Our findings suggest that the risk for NiV spillover to humans should be considered over a much wider area than previously regarded.
